# Coronavirus disease pandemic impact on emergency department visits for cardiovascular disease in Korea: A review

**DOI:** 10.1097/MD.0000000000035992

**Published:** 2023-11-24

**Authors:** Yeonjoo Cho, In Hwan Yeo, Dong Eun Lee, Jong Kun Kim

**Affiliations:** a Department of Emergency Medicine, Kyungpook National University Chilgok Hospital, School of Medicine, Kyungpook National University, Daegu, Republic of Korea.

**Keywords:** coronavirus disease pandemic, emergency department, fatality rate, myocardial infarction, stroke

## Abstract

The coronavirus disease (COVID-19) pandemic has affected patient visits to the hospital, including visits to the emergency department (ED). This study aimed to analyze the impact of the COVID-19 pandemic on the patterns of ED visits and treatment in hospitals for diseases requiring urgent diagnosis and treatment. We analyzed entries from the South Korea National Emergency Department Information System claims database between January 1, 2018 and December 31, 2020. We analyzed data of patients who visited the ED with acute myocardial infarction (AMI), acute ischemic stroke (AIS), and acute hemorrhagic stroke (AHS). We found that the COVID-19 pandemic had impacted ED utilization and fatality in patients with AMI, AIS, and AHS.

## 1. Introduction

The first case of coronavirus disease (COVID-19) was reported in Wuhan, China. Subsequently, the first case was reported in the Republic of Korea in January 2020 and has spread rapidly thereafter. On December 13, 2020, the number of confirmed cases in a day exceeded 1000 for the first time.^[1]^ The Korea has experienced 3 waves of the COVID-19 pandemic in 2020, and the Korean government responded COVID-19 pandemic with a policy of three-level social distancing that upscaled or downscaled depending on the number of COVID-19 patients.^[2]^ As the number of confirmed COVID-19 cases increased rapidly, older adults were waiting at home and were unable to receive proper treatment due to the lack of dedicated hospital beds.^[2,3]^

The COVID-19 pandemic has affected patient visits to the hospital, including visits to the emergency department (ED). This trend was also previously observed for both the severe acute respiratory syndrome and Middle East respiratory syndrome.^[4,5]^ Moreover, a study has shown that due to the COVID-19 pandemic, hospital visits of patients without COVID-19 during urgent medical situations decreased.^[6]^ Some studies have investigated the impact of COVID-19 on acute illnesses;^[7,8]^ however, only few were long-term nationwide studies.

Therefore, this study aimed to investigate the impact of the COVID-19 pandemic on the number of ED visits and fatality rate, as well as factors affecting fatality, in patients with acute myocardial infarction (AMI), acute ischemic stroke (AIS), and acute hemorrhagic stroke (AHS).

## 2. Methods

### 2.1. Study setting

The first case of COVID-19 in Korea was reported in January 2020; the number of cases has increased substantially thereafter. We defined 1 year from January 2020 as the main COVID-19 pandemic period and the 2 previous years (2018 and 2019) as the control period. We analyzed data of patients who visited the ED of the hospitals in Korea during the study period for the treatment of 3 major diseases, including AMI, AHS, and AIS.

### 2.2. Database and measurement variables

This study was a retrospective study that analyzed entries from the South Korea National Emergency Department Information System claims database between January 1, 2018 and December 31, 2020. The National Emergency Department Information System (NEDIS) database is a nationwide public database formed by the National Emergency Medical Center and contains all patient data who visited the ED of the hospitals in Korea. The database includes information on age, sex, region, disease onset time, ED arrival time, ED discharge time, ED visit route, ED discharge outcome, diagnosis codes according to the 10th revision of the International Classification of Diseases, and what medical services were used during stay in the ED. Three major diseases were defined based on the 10th revision of the International Classification of Diseases diagnostic code at discharge from the ED: AMI (I21–I22), AHS (I60–62), and AIS (I63–64). The outcomes were the number of ED visits, duration of symptom onset to ED arrival, length of ED stay, performance of emergency procedure, admission to the intensive care unit (ICU), and case fatality rate at the ED and hospital discharge.

Patients with AMI who underwent emergency procedures were defined as those who had undergone coronary angiography, percutaneous transluminal coronary angioplasty, stent insertion, thrombectomy, and off-pump coronary artery bypass surgery or with extracorporeal membrane oxygenation prescription code. Patients with AHS who underwent medical procedures were defined as those who had undergone craniotomy, craniectomy, burr hole, trephination, cerebral aneurysm clipping, and operation of cerebral arteriovenous malformation and embolization of cerebral aneurysm. Patients with AIS who underwent medical procedures were defined as those who had undergone intravenous thrombolysis, intra-arterial thrombolysis, extracranial percutaneous transluminal angioplasty or stent placement, and intracranial percutaneous transluminal angioplasty or stent placement.

All variables whose values were not entered, unknown, or missing were excluded. The duration of time to visit the ED after symptom onset and length of ED stay were calculated based on symptom onset time, ED visit time, and ED discharge time. The symptom onset of some patients was several months to several years before visiting the ED. To exclude chronic patients who could remarkably affect the statistics, the interquartile ranges were calculated, and the duration of ED visit after symptom onset exceeding the upper inner fences of the IQR (>Q3 + 1.5*IQR) was excluded.

### 2.3. Statistical analysis

All statistical analyses were performed using R version 4.1.0 (R Foundation for Statistical Computing, Vienna, Austria). Descriptive statistics, categorical variables, and continuous variables are expressed as number, percentage, and mean and standard deviation, or median (interquartile range) according to whether normal distributions or not. respectively. The chi-squared test was used to compare categorical variables between groups. Anderson-Darling normality test was performed to detect normality of continuous variables. Continuous variables without normality were statistically analyzed using the Mann–Whitney *U* test method between groups. Among the patients who visited the ED with AMI, AIS, and AHS, a multivariable logistic regression analysis was conducted to investigate associated factors with hospital death. Backward elimination method was used to select variables. Adjusted odds ratios and confidence intervals were calculated. Statistical significance was set at *P* < .05. A sensitivity analysis with the *E*-value to measure the robustness of the association between the COVID-19 pandemic and the outcomes for unmeasured or unadjusted confounding was performed.

### 2.4. Ethics statement

This study was approved by the Institutional Review Board of the Medicity Daegu (no. 2020-10-001). The requirement of written informed consent was waived due to the retrospective nature of the study and use of anonymized data.

## 3. Results

**Figure 1.**
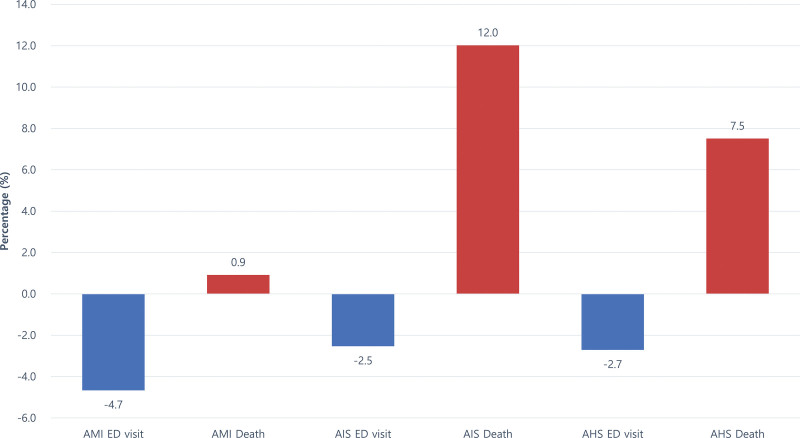
Changes in ED visits and fatality rates for cardiovascular and cerebrovascular disease during the COVID-19 pandemic relative to the control period. ED visit change: 200 × ED visit of 2020/(ED visit of 2018 + ED visit of 2019)–100. Change in deaths number: 200 × Deaths number of 2020/(Deaths number of 2018 + Deaths number of 2019) –100. AHS = acute hemorrhagic stroke, AIS = acute ischemic stroke, AMI = acute myocardial infarction, COVID-19 = coronavirus disease, ED = emergency department.

On comparing the average number of patients visiting the ED for AMI, AIS, and AHS before and during the pandemic, we found a decreasing trend at –4.7%, –2.5%, and –2.7%, respectively, however, the number of patients who died at the hospital increased to 0.9%, 12.0%, and 7.5%, respectively (Fig. 1).

The median length from symptom onset to ED visit increased from 144.0 minutes to 151.0 minutes for AMI (*P* < .001) and from 229.5 minutes to 240.0 minutes for AIS (*P* < .001). The median length of ED stays slightly increased from 194.0 minutes to 199.0 minutes for AHS (*P* = .002) (Table 1).

**Table 1:** Comparison between before and during the COVID-19 pandemic.

		AMI					AIS					AHS				
		Before COVID-19 pandemic (N = 60,576)		COVID-19 pandemic (N = 28,875)		*P*	Before COVID-19 pandemic (N = 160,121)		COVID-19 pandemic (N = 78,061)		*P*	Before COVID-19 pandemic (N = 56,254)		COVID-19 pandemic (N = 27,369)		*P*
Region	Seoul, Gyonggido, Incheon	27,467	45.3%	13,082	45.3%	.021	76,327	47.7%	36,419	46.7%	<.001	26,182	46.5%	12,330	45.1%	<.001
	Daegu, Gyeongsangbukdo	7042	11.6%	3186	11.0%		16,788	10.5%	7995	10.2%		5608	10.0%	2793	10.2%	
	Etc*	26,067	43.0%	12,607	43.7%		67,006	41.8%	33,647	43.1%		24,464	43.5%	12,246	44.7%	
Sex	Male	42,103	69.5%	20,348	70.5%	.003	90,212	56.3%	44,969	57.6%	<.001	29,080	51.7%	13,926	50.9%	.028
	Female	18,473	30.5%	8527	29.5%		69,909	43.7%	33,092	42.4%		27,174	48.3%	13,443	49.1%	
Age	≤19	57	0.1%	15	0.1%	.018	308	0.2%	123	0.2%	<.001	473	0.8%	201	0.7%	<.001
	20–74	40,242	66.4%	19,377	67.1%		91,970	57.4%	44,126	56.5%		39,825	70.8%	19,009	69.5%	
	≥75	20,277	33.5%	9483	32.8%		67,843	42.4%	33,812	43.3%		15,956	28.4%	8156	29.8%	
Transfer	Transfer	20,360	33.6%	8802	30.5%	<.001	36,930	23.1%	16,272	20.8%	<.001	18,113	32.2%	7621	27.8%	<.001
	Etc†	40,212	66.4%	20,072	69.5%		123,186	76.9%	61,787	79.2%		38,139	67.8%	19,746	72.2%	
Means of ED visit	Public EMS	20,646	34.1%	10,406	36.0%	<.001	6,1360	38.3%	3,1885	40.8%	<.001	26,758	47.6%	14,299	52.2%	<.001
	Private EMS	13,066	21.6%	5443	18.9%		2,1837	13.6%	9398	12.0%		14,966	26.6%	6217	22.7%	
	Etc‡	26,860	44.3%	13,024	45.1%		7,6922	48.0%	36,774	47.1%		14,527	25.8%	6852	25.0%	
Length of onset to ED visit (minute)		144.0 [59.0;420.0]		151.0 [60.0;434.0]		<.001	229.5 [68.0;837.0]		240.0 [74.0;848.5]		<.001	90.0 [40.0;240.0]		90.0 [44.0;240.0]		<.001
Length of ED stay (minute)		126.0 [57.0;252.0]		125.0 [56.0;253.0]		.616	188.0 [123.0;289.0]		188.0 [123.0;291.0]		.529	194.0 [125.0;300.0]		199.0 [127.0;305.0]		.002
Procedure	Non	23,421	38.7%	10,891	37.7%	.007	150,044	93.7%	71,796	92.0%	<.001	41,157	73.2%	19,576	71.5%	<.001
	Procedure	37,155	61.3%	17,984	62.3%		10,077	6.3%	6265	8.0%		15,097	26.8%	7793	28.5%	
Admission	ICU	24,447	52.9%	10,776	50.1%	<.001	25,230	22.5%	11,807	21.4%	<.001	22,522	55.7%	10,426	53.3%	<.001
	Etc§	21,738	47.1%	10,733	49.9%		86,994	77.5%	43,344	78.6%		17,899	44.3%	9126	46.7%	
ED discharge	Alive	59,105	97.6%	28,111	97.4%	.051	159,613	99.8%	77,760	99.8%	.119	55,579	98.8%	26,962	98.6%	.001
	Death	1437	2.4%	748	2.6%		341	0.2%	192	0.2%		655	1.2%	393	1.4%	
Hospital discharge	Alive	55,506	91.7%	26,318	91.2%	.015	154,396	96.5%	74,839	96.0%	<.001	49,349	87.8%	23,654	86.5%	<.001
	Death	5036	8.3%	2541	8.8%		5558	3.5%	3113	4.0%		6885	12.2%	3701	13.5%	

AHS = acute hemorrhagic stroke, AIS = acute ischemic stroke, AMI = acute myocardial infarction, COVID-19 = coronavirus disease, ED = emergency department, EMS = emergency medical service.

* All other regions except Seoul, Gyonggido, Incheon, Daegu, and Gyeongsangbukdo in Korea.

† It includes “direct visit” and “outpatient referral.”

‡ It includes public vehicles such as police cars, air transfers, other vehicles, and walking.

§ It includes hospitalizations in various wards except the ICU.

The proportion of patients with AMI, AIS, and AHS who were referred by other hospitals showed a decreasing trend. Regarding the ED visit routes, the rate of visits through public emergency medical service (EMS) increased, while the rate of visits through private EMS used for transferring patients from another hospital decreased. After visiting the hospital, the rate of all major procedures increased; however, the ICU admission rate decreased. The rate of death in the ED and after visiting the ED increased from 8.3% to 8.8% for AMI (*P* = .015), from 3.5% to 4.0% for AIS (*P* < .001), and from 12.2% to 13.5% for AHS (*P* < .001) (Table 1).

**Figure 2.**
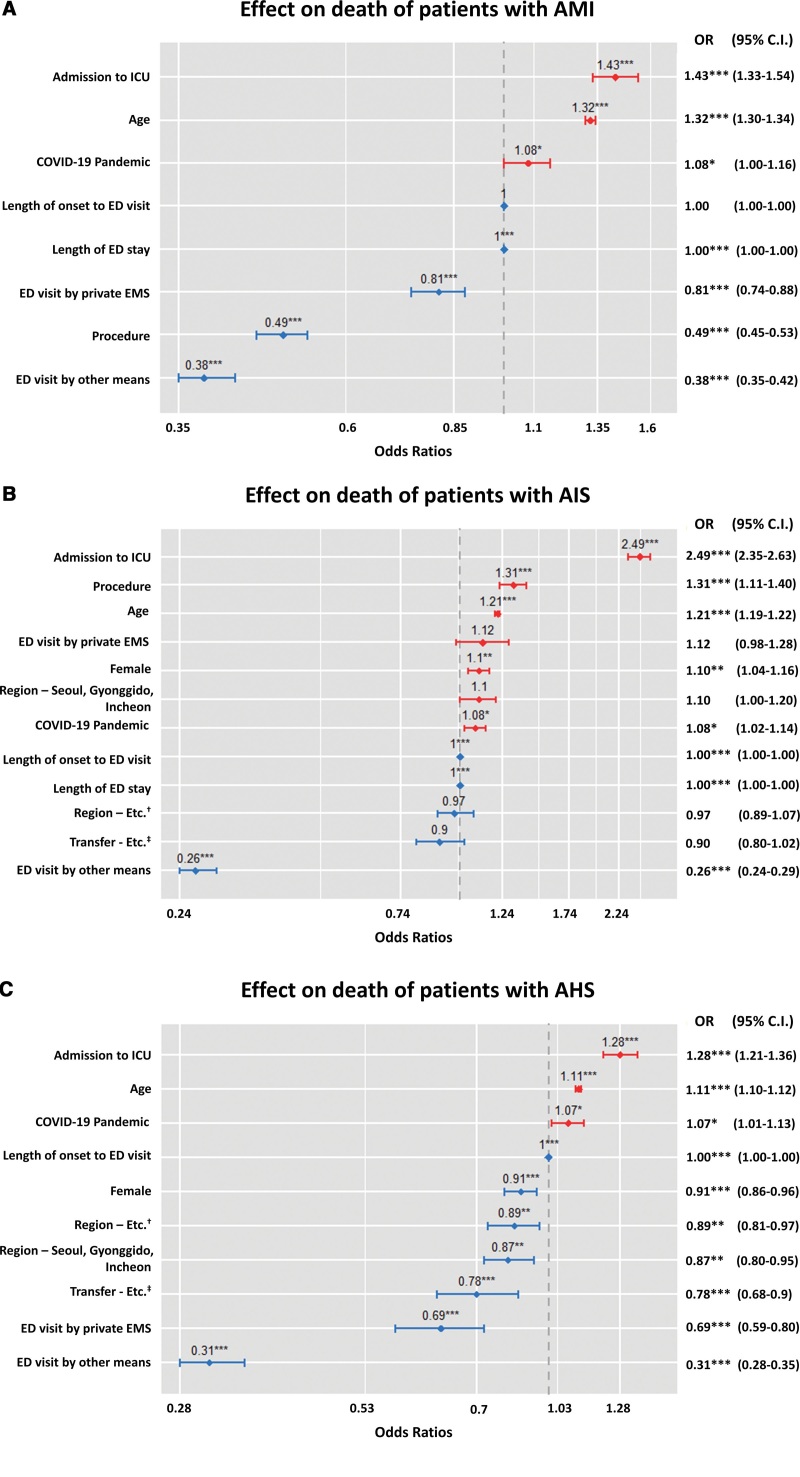
Effect on death of patients with AMI (A), AIS (B), and AHS (C). AHS = acute hemorrhagic stroke, AIS = acute ischemic stroke, AMI = acute myocardial infarction, C.I. = confidence intervals, COVID-19 = coronavirus disease, ED = emergency department, EMS = Emergency Medical Service, OR = Odds Ratios. ^*^P < .05. ^**^P < .01. ^***^P < .001. ^†^All other regions except Seoul, Gyonggido, Incheon, Daegu, and Gyeongsangbukdo in Korea. ^‡^It includes “direct visit” and ‘outpatient referral.

In the logistic regression analysis to find out the factors related to hospital death among patients who visited the ED with AMI, AIS, and AHS, COVID-19 pandemic was found to have an effect. The COVID-19 pandemic had impacted fatality in patients with AMI, AIS, and AHS (AMI: Odds Ratio [OR] 1.08, 95% confidence interval [CI] 1.00–1.16, *P* < .05, AIS: OR 1.08, CI 1.02–1.14, *P* < .05, AHS: OR 1.07, CI 1.01–1.13, *P* < .05). The E-value measured between the COVID-19 and AMI, AIS, and AHS were 1.37 (lower: 1), 1.37 (lower: 1.16), and 1.34 (lower: 1.11). However, the length from symptom onset to ED visit and the length of ED stay had no significant effect (Fig. 2).

## 4. Discussion

This is the first study that investigate the impact of the COVID-19 pandemic on ED visits among patients with cardiac and cerebrovascular problem who require prompt treatment during the COVID-19 pandemic.

The number of ED visits among patients with AMI, AIS and AHS significantly decreased since the outbreak of COVID-19 pandemic. Moreover, regarding ED visit routes, relative to the pre-pandemic period, the number of inter-hospital transfer and visits through private EMS decreased and use of 119 ambulances and self-visit increased. According to Centers for Disease Control and Prevention, the national hospital data showed that the number of ED visits in April 2020 compared to those a year ago decreased by approximately 42%, and it was evident in the case of low-severity diseases.^[9–11]^ A report from the United States showed that the number of ED visits decreased by 23% and 20% for heart attacks and strokes, respectively, in the 10 weeks following declaration of the COVID-19 national emergency.^[12]^ A French study found that hospitalizations for patients with ST-segment elevation myocardial infarction were reduced by approximately 18% compared to those before the COVID-19 pandemic.^[13]^ This might be attributed to a reduced total number of patients utilizing the ED. First, patients feared that contact with the health care system during hospital visits might increase their risk of contracting COVID-19. In a national survey conducted in April 2020, researchers found that >90% adults were concerned about contracting COVID-19 in the ED and that 29% adults responded that they avoided or delayed medical care because of anxiety of contracting COVID-19.^[14]^ Second, due to patient’s fear and confusion of their current symptoms as association with COVID-19, they might have underestimated the likelihood of a serious illness.^[15]^ Last, there might be a decision not to burden the emergency medical system, which was already exposed to heavy workload and shortage of medial resources. In addition, policies such as social distancing and expansion of COVID-19 testing for screening could have caused a decrease in hospital use.

Other studies have suggested that low-level physical activity and serious work-related stress during lockdown as part of social policy may have contributed to lowering the incidence of AMI.^[16,17]^ As the general ward and ED were closed due to confirmed COVID-19 cases and the number of patients showing symptoms of infection including fever and respiratory symptoms increased, the transfer between hospitals might have been limited. According to a local survey in Korea, EDs were repeatedly temporarily closed and an increase in medical staff undergoing self-quarantine was observed due to a surge in COVID-19 cases in February 2020.^[18]^

Moreover, we found that the time from symptom onset to visit to ED was delayed, and the time spent in the ED with AHS increased. There are several reasons for the delay in the time to ED visits. First, patients were reluctant to visit the hospital due to fear of COVID-19 transmission.^[19,20]^ Second, the number of ambulances available for patients was insufficient. Since patients with suspected COVID-19 stayed and were transported using ambulance, it could be a potential source of the disease.^[21]^ The use of ambulances might be restricted due to disinfection as it was used not only by confirmed patients but also by symptomatic individuals. According to some reports, the number of EMS calls increased rapidly during the COVID-19 pandemic, but the time required to answer calls was also increased significantly.^[22,23]^ The turn-around time for confirming results of COVID-19 in acute-stage patients might have influenced the increase in the length of stay in the ED. As each hospital required confirmation of the COVID-19 test result as a system for admission, patients might have stayed longer in the ED until they were confirmed to be negative for COVID-19.

However, the rate of procedures including interventions such as coronary angiography and reperfusion increased compared to that before the pandemic. In addition, the rate of admission to the ICU decreased, and the fatality rate at the time of discharge from the ED and general ward increased. According to the European Society of Cardiology Guidelines, when ST-segment elevation myocardial infarction is diagnosed, immediate percutaneous coronary intervention is necessary because the fatality rate increases if treatment is delayed.^[24]^ In stroke, hyper-acute treatment such as thrombolysis and thrombectomy also affects the neurological outcome and fatality of patients.^[25]^ Although emergency procedures were performed, timely procedures could be delayed due to time delay after symptom onset, and the ED waiting time and admission rate to the ICU might have affected the fatality rate.

This study has several limitations. First, there is a lack of the detailed process for diagnosis and treatment using the NEDIS data compared with an in-depth review of hospital records. Second, as a limitation of the NEDIS data, there is a lack of analysis of factors other than those related to COVID-19 affecting ED visits, such as patient’s classification systems, procedures, and hospitalization indications in each hospital. Third, there is a lack of the evaluation in the study about other factors that may affect the relationship between the variables. Last, an analysis of the direct impact of COVID-19 on each severe disease is lacking.

## 5. Conclusion

During the COVID-19 pandemic, the fatality rate of patients with cardiovascular diseases, such as AMI, AIS, and AHS, visiting the ED has increased. We hypothesized that the reduction of ED utilization due to the fear of contracting the disease, reduced capacity for transporting patients to the ED, increased difficulty in transporting patients to another hospital, increased length of ED stay, and decreased ICU admission after ED visit might have impacted the increase in fatality rate of patients with acute illnesses. To reduce fatality, effort should be made to improve these conditions.

## Acknowledgments

We would like to thank Editage (www.editage.co.kr) for English language editing.

## Author contributions

**Conceptualization:** Yeonjoo Cho, In Hwan Yeo, Dong Eun Lee, Jong Kun Kim.

**Methodology:** In Hwan Yeo.

**Supervision:** Jong Kun Kim, In Hwan Yeo.

**Validation:** Yeonjoo Cho.

**Writing – original draft:** Yeonjoo Cho, In Hwan Yeo.

**Writing – review & editing:** Yeonjoo Cho, In Hwan Yeo, Dong Eun Lee.

## References

[1] Prevention KCfDca. Current status of COVID-19 outbreak in Korea (December 13). 2020. Available at: http://ncov.mohw.go.kr/tcmBoardView.do?brdId=&brdGubun=&dataGubun=&ncvContSeq=361637&contSeq=361637&board_id=140&gubun=BDJ. [access July 28, 2022].

[2] Seong H Hyun HJ Yun JG. Comparison of the second and third waves of the COVID-19 pandemic in South Korea: importance of early public health intervention. Int J Infect Dis. 2021;104:742–5. 33556610 10.1016/j.ijid.2021.02.004 PMC7863747

[3] Prevention KCfDca. Regular briefing of the central disaster and safety countermeasures headquarters on COVID-19 outbreak. 2020. Available at: http://ncov.mohw.go.kr/tcmBoardView.do?brdId=&brdGubun=&dataGubun=&ncvContSeq=362027&contSeq=362027&board_id=&gubun=ALL. [Accessed July 28, 2022].

[4] Chen WK Cheng YC Chung YT. The impact of the SARS outbreak on an urban emergency department in Taiwan. Med Care. 2005;43:168–72. 15655430 10.1097/00005650-200502000-00010

[5] Lee SY Khang YH Lim HK. Impact of the 2015 Middle East respiratory syndrome outbreak on emergency care utilization and mortality in South Korea. Yonsei Med J. 2019;60:796–803. 31347336 10.3349/ymj.2019.60.8.796 PMC6660446

[6] Jeffery MM D’Onofrio G Paek H. Trends in emergency department visits and hospital admissions in health care systems in 5 states in the first months of the COVID-19 pandemic in the US. JAMA Intern Med. 2020;180:1328–33. 32744612 10.1001/jamainternmed.2020.3288 PMC7400214

[7] Sung HK Paik JH Lee YJ. Impact of the COVID-19 outbreak on emergency care utilization in patients with acute myocardial infarction: a nationwide population-based study. J Korean Med Sci. 2021;36:e111. 33904263 10.3346/jkms.2021.36.e111 PMC8076842

[8] Seo AR Lee WJ Woo SH. Pre-hospital delay in patients with acute stroke during the initial phase of the coronavirus disease 2019 outbreak. J Korean Med Sci. 2022;37:e47. 35166083 10.3346/jkms.2022.37.e47 PMC8845098

[9] Hartnett KP Kite-Powell A DeVies J. Impact of the COVID-19 pandemic on emergency department visits – United States, January 1, 2019–May 30, 2020. MMWR Morb Mortal Wkly Rep. 2020;69:699–704. 32525856 10.15585/mmwr.mm6923e1 PMC7315789

[10] Lucero AD Lee A Hyun J. Underutilization of the emergency department during the COVID-19 pandemic. West J Emerg Med. 2020;21:15–23. 33052821 10.5811/westjem.2020.8.48632 PMC7673895

[11] Butt AA Azad AM Kartha AB. Volume and acuity of emergency department visits prior to and after COVID-19. J Emerg Med. 2020;59:730–4. 32919838 10.1016/j.jemermed.2020.08.013 PMC7413056

[12] Lange SJ Ritchey MD Goodman AB. Potential indirect effects of the COVID-19 pandemic on use of emergency departments for acute life-threatening conditions – United States, January–May 2020. Am J Transplant. 2020;20:2612–7. 32862556 10.1111/ajt.16239 PMC9800659

[13] Rangé G Hakim R Motreff P. Where have the ST-segment elevation myocardial infarctions gone during COVID-19 lockdown? Eur Heart J Qual Care Clin Outcomes. 2020;6:223–4. 32348457 10.1093/ehjqcco/qcaa034 PMC7197594

[14] Physicians ACoE. Public poll: emergency care concerns amidst COVID-19. 2020. Available at: https://www.emergencyphysicians.org/article/covid19/public-poll-emergency-care-concerns-amidst-covid-19. [Accessed July 28, 2022].

[15] Hammad TA Parikh M Tashtish N. Impact of COVID-19 pandemic on ST-elevation myocardial infarction in a non-COVID-19 epicenter. Catheter Cardiovasc Interv. 2021;97:208–14. 32478961 10.1002/ccd.28997 PMC7300525

[16] Claeys MJ Argacha JF Collart P. Impact of COVID-19-related public containment measures on the ST elevation myocardial infarction epidemic in Belgium: a nationwide, serial, cross-sectional study. Acta Cardiol. 2021;76:863–9. 32727305 10.1080/00015385.2020.1796035

[17] Danchin N Marijon E. COVID-19 pandemic: preventing hospital myocardial infarction admissions or preventing acute myocardial infarction altogether? Heart. 2021;107:436–7. 10.1136/heartjnl-2020-318646 33526507

[18] Chung HS Lee DE Kim JK. Revised triage and surveillance protocols for temporary emergency department closures in tertiary hospitals as a response to COVID-19 crisis in Daegu metropolitan city. J Korean Med Sci. 2020;35:e189. 32419401 10.3346/jkms.2020.35.e189 PMC7234857

[19] Barten DG Latten GH Van Osch FH. Reduced emergency department utilization during the early phase of the COVID-19 pandemic: viral fear or lockdown effect? Disaster Med Public Health Prep. 2022;16:36–9. 32782063 10.1017/dmp.2020.303 PMC7503047

[20] Barten D Latten G. Re:“Non-COVID-19 visits to emergency departments during the pandemic: the impact of fear”. Public Health. 2020;185:47. 32540610 10.1016/j.puhe.2020.05.063 PMC7275175

[21] Makiela S Taylor-Robinson A Weber A. A preliminary assessment of contamination of emergency service helicopters with MRSA and multi-resistant Staphylococcus aureus. Emergency Medicine. 2016;6:304.

[22] Semeraro F Gamberini L Tartaglione M. An integrated response to the impact of coronavirus outbreak on the Emergency Medical Services of Emilia Romagna. Resuscitation. 2020;151:1–2. 32205158 10.1016/j.resuscitation.2020.03.005 PMC7118616

[23] Lapostolle F Agostinucci JM Alhéritière A. Collateral consequences of COVID-19 epidemic in Greater Paris. Resuscitation. 2020;151:6–7. 32283116 10.1016/j.resuscitation.2020.04.010 PMC7195282

[24] Ibanez B James S Agewall S. 2017 ESC guidelines for the management of acute myocardial infarction in patients presenting with ST-segment elevation: the task force for the management of acute myocardial infarction in patients presenting with ST-segment elevation of the European Society of Cardiology (ESC). Eur Heart J. 2017;39:119–77. 10.1093/eurheartj/ehx393 28886621

[25] Khosravani H Rajendram P Notario L. Protected code stroke: Hyperacute stroke management during the coronavirus disease 2019 (COVID-19) pandemic. Stroke. 2020;51:1891–5. 32233980 10.1161/STROKEAHA.120.029838 PMC7258750

